# Long-term visit-to-visit glycemic variability as predictor of micro- and macrovascular complications in patients with type 2 diabetes: The Rio de Janeiro Type 2 Diabetes Cohort Study

**DOI:** 10.1186/s12933-018-0677-0

**Published:** 2018-02-24

**Authors:** C. R. L. Cardoso, N. C. Leite, C. B. M. Moram, G. F. Salles

**Affiliations:** 1 Department of Internal Medicine, University Hospital Clementino Fraga Filho, School of Medicine, Universidade Federal do Rio de Janeiro, Rua Croton, 72, Jacarepagua, Rio de Janeiro, RJ CEP: 22750-240 Brazil; 2 Department of Occupational Therapy, University Hospital Clementino Fraga Filho, School of Medicine, Universidade Federal do Rio de Janeiro, Rio de Janeiro, Brazil

**Keywords:** Glycemic variability, Macrovascular complications, Microvascular complications, Mortality, Type 2 diabetes

## Abstract

**Background:**

Long-term visit-to-visit glycemic variability is an additional measure of glycemic control. We aimed to evaluate the prognostic value of several measures of glycemic variability for the occurrence of micro- and macrovascular complications, and all-cause mortality in patients with type 2 diabetes.

**Methods:**

654 individuals were followed-up over a median of 9.3 years. Glycemic variability (SDs and coefficients of variation of HbA_1c_ and fasting glycaemia) was measured during the first 12- and 24-months. Multivariate Cox analysis, adjusted for risk factors and mean HbA_1c_ and fasting glycaemia levels, examined the associations between glycemic variability and the occurrence of microvascular (retinopathy, microalbuminuria, renal function deterioration, peripheral neuropathy) and macrovascular complications [total cardiovascular events (CVE), major adverse CVEs (MACE) and cardiovascular mortality], and of all-cause mortality.

**Results:**

During follow-up, 128 patients had a CVE (96 MACE), and 158 patients died (67 from cardiovascular diseases); 152 newly-developed or worsened diabetic retinopathy, 183 achieved the renal composite outcome (89 newly developed microalbuminuria and 91 deteriorated renal function), and 96 newly-developed or worsened peripheral neuropathy. Glycemic variability, particularly the 24-month parameters either estimated by HbA_1c_ or by fasting glycemia, predicted all endpoints, except for retinopathy and peripheral neuropathy development/progression, and was a better predictor than mean HbA_1c_. Glycemic variability predicted retinopathy development/progression in patients with good glycemic control (HbA_1c_ ≤ 7.5%, 58 mmol/mol) and predicted new-incident peripheral neuropathy.

**Conclusions:**

Long-term visit-to-visit glycemic variability is an additional and frequently a better glycemic parameter than mean HbA_1c_ levels for assessing the risk of future development of micro- and macrovascular complications in patients with type 2 diabetes.

## Background

Type 2 diabetes is an important public health problem worldwide, not only by its increasing prevalence, but also because its associated micro- and macrovascular complications severely impact on individuals’ quality of life and pose a great burden on healthcare systems [1, 2]. Current diabetes treatment aims to reducing chronic complications development and progression, mainly by controlling hyperglycemia, high blood pressure (BP) levels and dyslipidemia; and glycemic control is traditionally monitored by serial mean glycated hemoglobin (HbA_1c_) levels [3]. This rationale comes from several trials and observational studies showing that lowering HbA_1c_ indeed reduces the risk of micro- and macrovascular complications [4–6]. However, particularly for macrovascular disease, there is no consensus that lowering HbA1c to levels below 8.5–8.0% (69–64 mmol/mol) actually reduces such risk [7, 8]. Moreover, even for microvascular complications there may be additional glycemic factors associated with increased risks, beyond mean HbA1c levels [9, 10]. In this context, the concept of glycemic variability has recently emerged as another measure of glycemic control, which might constitute an additive, or even better predictor of diabetic complications than mean HbA1c levels [11, 12].

Glycemic variability is a general denomination to several measures of short-term and long-term fluctuations in glycemia. Short-term glycemic variability refers to within-day or between-days glycemia fluctuations, and is usually measured by continuous glucose monitoring mainly in individuals with type 1 diabetes [12]. Long-term glycemic variability refers to glycemic fluctuations over months to years and is generally measured by visit-to-visit variability in either HbA_1c_ or fasting glycemia (FG) in both type 1 and type 2 diabetes [12]. Several previous studies in patients with type 2 diabetes [13–41] showed that long-term glycemic variability may predict both microvascular (retinopathy [13–18], diabetic kidney disease [16, 18–26], and cardiovascular autonomic neuropathy [27]) and macrovascular complications [23, 28–36], as well as all-cause mortality [29, 32–34, 36–41]. However, there were conflicting results, particularly regarding diabetic retinopathy [14, 16–18] and cardiovascular disease incidence [33–36]. Moreover, except for a few reports [16, 18, 23, 29, 32–34, 36], most of the previous studies evaluated only a single diabetic complication, without a more comprehensive analysis of several adverse diabetic complications. Only a recent meta-analysis [12] provided such extensive examination. Finally, the value of glycemic variability as a predictor of future diabetic peripheral neuropathy development and progression, an important microvascular complication, as far as we know, has not been evaluated yet. In resume, the importance of long-term visit-to-visit glycemic variability measures as predictors of future complications in patients with type 2 diabetes still remains controversial [42, 43], and further studies are needed.

Therefore, the aim of this study was to evaluate the prognostic value of several measures of long-term glycemic variability for the occurrence of separated microvascular (retinopathy, microalbuminuria, renal function deterioration, and peripheral neuropathy) and macrovascular complications (total cardiovascular events, major cardiovascular events, cardiovascular mortality), and all-cause mortality in The Rio de Janeiro Type 2 Diabetes (RIO-T2D) Cohort Study, an on-going cohort of high cardiovascular risk individuals with type 2 diabetes with a median follow-up of nearly 10 years.

## Methods

### Patients and baseline procedures

This was a prospective study, nested within The Rio de Janeiro Type 2 Diabetes Cohort Study, with 654 patients with type 2 diabetes enrolled between August 2004 and December 2008 and re-evaluated annually until December 2016 in the diabetes outpatient clinic of our tertiary-care University Hospital. All participants gave written informed consent, and the local Ethics Committee had previously approved the study protocol. The characteristics of this cohort, the baseline procedures and the diagnostic definitions have been detailed elsewhere [44–47]. In brief, inclusion criteria were all adult type 2 diabetic individual up to 80 years old with either any microvascular (retinopathy, nephropathy or neuropathy) or macrovascular (coronary, cerebrovascular or peripheral artery disease) complication, or with at least two other modifiable cardiovascular risk factors. Exclusion criteria were morbid obesity (body mass index ≥ 40 kg/m^2^), advanced renal failure (serum creatinine > 180 μmol/l or estimated glomerular filtration rate < 30 ml/min/1.73 m^2^) or the presence of any serious concomitant disease limiting life expectancy. All were submitted to a standard baseline protocol that included a thorough clinical examination, a laboratory evaluation, and a 24-h ambulatory BP monitoring (ABPM). Diagnostic criteria for diabetic chronic complications were detailed previously [44–47]. In brief, coronary heart disease was diagnosed by clinical, electrocardiographic criteria, or by positive ischemic stress tests. Cerebrovascular disease was diagnosed by history and physical examination, and peripheral arterial disease by an ankle-brachial index < 0.9. The diagnosis of nephropathy needed at least two albuminurias ≥30 mg/24 h or proteinurias ≥0.5 g/24 h or confirmed reduction of glomerular filtration rate (eGFR ≤ 60 ml/min/1.73 m^2^, estimated by the CKD-EPI equation, or serum creatinine > 130 μmol/l). Peripheral neuropathy was determined by clinical examination (knee and ankle reflex activities, feet sensation with the Semmes–Weinstein monofilament, vibration with a 128-Hz tuning fork, pinprick and temperature sensations) and neuropathic symptoms were assessed by a standard validated questionnaire [45]. Clinic blood pressure (BP) was measured three times using a digital oscillometric BP monitor (HEM-907XL, Omron Healthcare, Kyoto, Japan) with a suitable sized cuff on two occasions 2 weeks apart at study entry. The first measure of each visit was discarded and BP considered was the mean between the last two readings of each visit. Arterial hypertension was diagnosed if mean systolic (SBP) ≥ 140 mmHg or diastolic BP (DBP) ≥ 90 mmHg or if anti-hypertensive drugs had been prescribed. ABPM was recorded in the following month using Mobil-O-Graph, version 12 equipment (Dynamapa, Cardios LTDA., São Paulo, Brazil), and average 24-h SBP and DBP were registered [47]. Laboratory evaluation included fasting glycemia (FG), glycated hemoglobin (HbA_1c_), serum creatinine and lipids. Albuminuria and proteinuria were evaluated in two non-consecutive sterile 24-h urine collections.

### Long-term glycemic variability measurements

The patients had at least three annual HbA_1c_ and FG measurements during follow-up. Long-term visit-to-visit glycemic variability was estimated separately for HbA_1c_ and FG, and for the first 12 and 24-month periods, as the standard deviation (SD) of all measurements performed during these periods. To account for the possible influence of different number of measurements, the SD was divided by $\sqrt {[n} /\left( {n - 1} \right)]$, as previously suggested [10]. The coefficient of variation (SD/mean) was also calculated for each glycemic variability parameter.

### Follow-up and outcomes assessment

The patients were followed-up regularly at least 3–4 times a year until December 2016 under standardized treatment. The observation period for each patient was the number of months from the date of the first clinical examination to the date of the last clinical visit in 2016 or the date of the first endpoint, whichever came first. The primary endpoints were the occurrence of any macrovascular or microvascular outcomes. Macrovascular outcomes were total cardiovascular events (CVEs: fatal or non-fatal myocardial infarctions, sudden cardiac deaths, new-onset heart failure, death from progressive heart failure, any myocardial revascularization procedure, fatal or non-fatal strokes, any aortic or lower limb revascularization procedure, any amputation above the ankle, and deaths from aortic or peripheral arterial disease), major adverse cardiovascular events (MACE: non-fatal myocardial infarctions and strokes plus cardiovascular deaths), and all-cause and cardiovascular mortalities [44]. Mortality, as well as non-fatal cardiovascular events occurrence, was ascertained from medical records, death certificates and interviews with attending physicians and patient families, by a standard questionnaire reviewed by two independent observers. In case of disagreement, it was decided by consensus with a third independent consultant. Most of the in-hospital fatal or non-fatal events were attended at our own hospital. Microvascular outcomes were retinopathy development or worsening [46], renal outcomes [47] [new microalbuminuria development, new renal failure development (defined as doubling of serum creatinine or end-stage renal failure needing dialysis or death from renal failure), and a composite of them], and peripheral neuropathy development or worsening [45]. Retinopathy and renal outcomes were evaluated by annual examinations [46, 47], whereas peripheral neuropathy was evaluated on a second specific examination performed after a median of 6 years from the baseline examination [45].

### Statistical analyses

Continuous data were described as means (SD) or as medians (interquartile range). For initial exploratory analyses, patients were categorized into tertiles of glycemic variability parameters and baseline characteristics compared by ANOVA, Kruskal–Wallis or χ^2^ tests, when appropriate. Kaplan–Meier curves of cumulative endpoints incidence during follow-up, compared by log-rank tests, were used for assessing different incidences of outcomes among tertile subgroups. For assessing the prognostic value of each glycemic variability parameter for each macrovascular and microvascular outcome, except for peripheral neuropathy, a time-to-event Cox analysis was undertaken with progressively increasing statistical adjustments for potential confounding. Model 1 was only adjusted for age, sex and number of HbA_1c_ or FG measurements, model 2 was further adjusted for other potential confounders (diabetes duration, body mass index (BMI), smoking status, physical inactivity, arterial hypertension, number of anti-hypertensive drugs in use, ambulatory 24-h SBP, presence of each micro- and macrovascular complications at baseline, serum mean HDL- and LDL-cholesterol, and use of insulin, statins and aspirin), and model 3 was further adjusted for mean FG and HbA_1c_ levels during the same period of glycemic variability measurement. These results were presented as hazard ratios (HRs) with their 95% confidence intervals (CIs); to allow comparisons among different glycemic variability parameters, their HRs were calculated for standardized increments of 1-SD. As glycemic variability was measured during the first 2 years of follow-up, patients who presented any of the endpoints during this period were excluded from the analysis of this specific outcome. For peripheral neuropathy analyses, a multiple logistic regression was used with the same progressively increasing statistical adjustments, except that height (instead of BMI) and the time interval between the baseline and second neuropathy evaluations were included as adjusting covariates. These results were reported as odds ratios (ORs) with their respective 95% CIs, also estimated for increments of 1-SD in each glycemic variability parameter. The same analyses were performed for patients categorized into tertiles of each glycemic variability parameter, with HRs and ORs calculated for the highest tertile subgroup in relation to the lowest tertile reference subgroup, after adjustments for the same covariates. Interaction between mean HbA_1c_ and glycemic variability measures were tested for all endpoints and whenever there was evidence of interaction (p < 0.10 for interaction term), stratified analyses for high (> 7.5%, 58 mmol/mol) and low (≤ 7.5%) HbA_1c_ levels were performed. Statistics were performed with SPSS version 19.0 (SPSS Inc, Chicago, Il., USA), and a 2-tailed probability value < 0.05 was considered significant.

## Results

### Baseline characteristics

For macrovascular and mortality outcomes, 654 patients without any endpoint occurrence during the first 2 years of follow-up were evaluated. For microvascular outcomes, 615 patients were evaluated for renal, 533 for retinopathy and 471 for peripheral neuropathy outcomes. Patients had a median of 4 HbA_1c_ (range 3–6) and 5 FG (range 3–7) measurements during the first 12 months of follow-up, and a median of 8 HbA_1c_ (range 6–11) and 10 FG (range 6–14) measurements during the first 24 months of follow-up. Median time interval between each visit-to-visit HbA_1c_ and FG measurements was 3 months. Table 1 outlines the baseline characteristics of all patients and of those divided according to tertiles of 24-month HbA_1c_ variability. Patients with higher HbA_1c_-SD were younger, but with longer diabetes duration, and had higher prevalences of microvascular complications than those with lower HbA_1c_ variability. They also had higher BP levels, particularly at ABPM, and poorer glycemic control, although using insulin more frequently, than those with lower HbA_1c_ variability. Table 2 shows the same baseline characteristics of patients divided according to 24-month FG variability. In general, they follow the same patterns of HbA_1c_ variability, except that patients with higher FG-SD also had greater prevalences of macrovascular complications than those with lower FG variability.

**Table 1:** Characteristics of all diabetic patients and divided into tertiles of 24-month HbA_1c_ variability

Characteristics	All patients (n = 654)	1st-tertile HbA_1c_-SD ≤ 0.45% (n = 218)	2nd-tertile HbA_1c_-SD 0.46–0.85% (n = 218)	3rd-tertile HbA_1c_-SD ≥ 0.86% (n = 218)	p value
Age (years)	60.1 (9.6)	61.2 (9.4)	59.9 (9.9)	58.7 (9.4)	0.007
Male sex (%)	38.1	42.2	37.6	34.4	0.26
BMI (kg/m^2^)	29.7 (4.8)	29.9 (4.6)	29.7 (5.0)	29.6 (5.0)	0.84
Smoking, current/past (%)	45.1	48.2	40.8	46.3	0.29
Physical activity (%)	22.4	23.4	23.5	20.2	0.64
Diabetes duration (years)	8.0 (3.0–15.0)	5.0 (1.4–12.3)	10.0 (4.0–17.5)	9.0 (5.0–15.0)	< 0.001
Chronic diabetic complications (%)					
Cerebrovascular disease	9.0	11.5	5.5	10.1	0.075
Coronary artery disease	15.6	14.7	15.1	17.0	0.83
Peripheral artery disease	17.0	15.7	17.1	18.3	0.76
Retinopathy	32.7	27.1	34.1	36.7	0.087
Nephropathy	31.0	22.0	27.3	43.6	< 0.001
Peripheral neuropathy	29.0	26.5	31.2	29.2	0.56
Cardiovascular autonomic neuropathy	18.1	14.9	17.4	21.8	0.24
Diabetes treatment (%)					
Metformin	87.9	89.4	86.2	88.1	0.61
Sulfonylureas	43.3	44.5	43.6	41.7	0.86
Insulin	48.9	29.8	51.8	65.1	< 0.001
Aspirin	90.9	91.2	87.6	94.0	0.068
Dyslipidemia (%)	87.3	87.2	86.7	88.1	0.93
Statins use (%)	77.5	79.7	75.6	77.1	0.57
Arterial hypertension (%)	86.5	84.4	88.1	87.2	0.51
Number of anti-hypertensive drugs	3 (1–4)	3 (1–4)	3 (1–4)	3 (1–4)	0.97
ACE inhibitors/AR blockers (%)	83.0	82.0	82.0	85.0	0.65
Diuretics (%)	67.7	68.4	66.5	68.1	0.92
Calcium channel blockers (%)	31.7	35.4	33.0	26.6	0.14
Beta-blockers (%)	50.1	47.6	51.0	51.7	0.68
Blood pressures (mmHg)					
Clinic SBP	147 (25)	147 (25)	145 (25)	149 (24)	0.12
Clinic DBP	84 (13)	83 (13)	84 (13)	86 (14)	0.072
Ambulatory 24 h SBP	128 (15)	126 (15)	128 (15)	131 (16)	0.002
Ambulatory 24 h DBP	74 (10)	72 (9)	74 (10)	75 (11)	0.009
Laboratory variables					
Baseline FG (mmol/l)	8.97 (3.86)	7.53 (2.54)	8.83 (3.40)	10.54 (4.71)	< 0.001
Mean 12-month FG	8.10 (2.42)	6.90 (1.46)	7.98 (2.04)	9.35 (2.78)	< 0.001
Mean 24-month FG	8.09 (2.39)	6.93 (1.47)	7.97 (2.07)	9.35 (2.78)	< 0.001
Baseline HbA_1c_ (%)	8.1 (1.9)	7.0 (1.3)	8.0 (1.6)	9.1 (2.1)	< 0.001
(mmol/mol)	65 (20.8)	53 (14.2)	64 (17.5)	76 (23.0)	
Mean 12-month HbA_1c_ (%)	7.8 (1.5)	6.8 (0.7)	7.7 (1.2)	9.0 (1.4)	< 0.001
(mmol/mol)	62 (16.4)	51 (7.7)	61 (13.1)	75 (15.3)	
Mean 24-month HbA_1c_ (%)	7.8 (1.4)	6.8 (0.8)	7.7 (1.2)	9.0 (1.4)	< 0.001
(mmol/mol)	62 (15.3)	51 (8.7)	61 (13.1)	75 (15.3)	
Triacylglycerol (mmol/l)	2.0 (1.5)	1.8 (1.2)	2.0 (1.6)	2.2 (1.6)	0.064
HDL-cholesterol (mmol/l)	1.11 (0.30)	1.10 (0.27)	1.12 (0.33)	1.11 (0.32)	0.79
LDL-cholesterol (mmol/l)	3.03 (1.00)	2.96 (0.91)	3.00 (1.08)	3.13 (0.99)	0.20
Glomerular filtration rate (ml/min/1.73 m^2^)	81 (20)	80 (19)	82 (20)	80 (22)	0.53
Albuminuria (mg/24 h)	13 (7–42)	10 (6–22)	13 (7–41)	19 (8–90)	< 0.001
Macrovascular outcomes^a^					
Total CVEs	128 (2.56)	36 (2.20)	34 (1.99)	58 (3.67)	0.005
Major CVEs	96 (1.86)	24 (1.42)	24 (1.38)	48 (2.93)	0.001
Cardiovascular mortality	67 (1.26)	16 (0.92)	15 (0.85)	36 (2.07)	0.002
All-cause mortality	158 (2.97)	44 (2.52)	49 (2.77)	65 (3.74)	0.10
Microvascular outcomes^b^					
Retinopathy (incident/worsening) (n = 533)	152 (4.88)	30 (2.63)	50 (4.89)	72 (7.96)	< 0.001
Renal composite (n = 615)	183 (4.71)	54 (4.11)	47 (3.63)	82 (6.58)	< 0.001
Microalbuminuria (incident) (n = 436)	89 (3.23)	31 (2.88)	25 (2.80)	33 (4.26)	0.21
Renal failure (n = 615)	91 (2.15)	19 (1.35)	24 (1.72)	48 (3.42)	< 0.001
Peripheral neuropathy (incident/worsening) (n = 471)	96 (20.4%)	21 (13.5%)	35 (22.0%)	40 (25.5%)	0.011
Peripheral neuropathy (incident) (n = 338)	42 (12.4%)	11 (9.4%)	9 (8.2%)	22 (19.8%)	0.005

Values are proportions, and means (standard deviations) or medians (interquartile range)

*HbA*_*1c*_ glycated hemoglobin, *ACE* angiotensin-converting enzyme, *AR* angiotensin II receptor, *SBP* systolic blood pressure, *DBP* diastolic blood pressure, *FG* fasting glycemia, *HDL* high-density lipoprotein, *LDL* low-density lipoprotein, *CVEs* cardiovascular events

^a^Values are absolute numbers (incidence rate per 100 patient-years of follow-up)

^b^Values are absolute numbers (incidence rate per 100 patient-years of follow-up), except for peripheral neuropathy that are absolute numbers (proportions)

**Table 2:** Characteristics and endpoints incidence of all diabetic patients and divided into tertiles of 24-month fasting glycemia variability

Characteristics	All patients (n = 654)	1st-tertile FG-SD ≤ 1.40 mmol/l (n = 218)	2nd-tertile FG-SD 1.41–2.60 mmol/l (n = 218)	3rd-tertile FG-SD ≥ 2.61 mmol/l (n = 218)	p value
Age (years)	60.1 (9.6)	60.6 (9.8)	60.5 (9.2)	59.1 (9.5)	0.16
Male sex (%)	38.1	38.7	36.2	39.9	0.45
BMI (kg/m^2^)	29.8 (4.6)	29.9 (4.6)	29.9 (5.0)	29.5 (5.0)	0.63
Smoking, current/past (%)	45.1	46.5	38.5	50.9	0.031
Physical activity (%)	22.4	27.6	20.2	18.9	0.017
Diabetes duration (years)	8 (3–15)	4 (1–10)	10 (5–15)	10 (5–17)	< 0.001
Chronic diabetic complications (%)					
Cerebrovascular disease	9.0	6.9	6.4	13.3	0.019
Coronary artery disease	15.6	11.1	15.1	21.1	0.015
Peripheral artery disease	17.0	10.6	19.9	20.6	0.008
Retinopathy	32.7	19.2	36.4	41.4	< 0.001
Nephropathy	31.0	22.4	26.0	45.8	< 0.001
Peripheral neuropathy	29.0	20.4	30.4	37.3	0.001
Cardiovascular autonomic neuropathy	18.1	12.4	21.2	21.4	0.042
Diabetes treatment (%)					
Metformin	87.9	90.8	91.3	80.7	0.001
Sulfonylureas	43.3	40.6	50.9	39.4	0.029
Insulin	48.9	24.9	52.3	68.8	< 0.001
Aspirin	90.9	87.5	90.2	94.5	0.040
Dyslipidemia (%)	87.3	88.0	87.2	86.7	0.92
Statins use (%)	77.5	78.2	77.9	76.0	0.84
Arterial hypertension (%)	86.5	83.9	89.0	86.6	0.29
Number of anti-hypertensive drugs	3 (1–4)	2 (1–3)	3 (2–4)	3 (1–4)	0.001
ACE inhibitors/AR blockers (%)	83.0	80.6	84.2	84.8	0.47
Diuretics (%)	67.7	59.7	75.7	68.6	0.002
Calcium channel blockers (%)	31.7	26.7	35.6	34.3	0.11
Beta-blockers (%)	50.1	43.2	54.0	53.9	0.043
Blood pressures (mmHg)					
Clinic SBP	147 (25)	143 (22)	149 (26)	149 (25)	0.012
Clinic DBP	84 (13)	83 (12)	85 (14)	85 (14)	0.11
Ambulatory 24 h SBP	128 (15)	125 (13)	129 (15)	131 (17)	0.001
Ambulatory 24 h DBP	74 (10)	73 (9)	74 (9)	75 (11)	0.078
Laboratory variables					
Baseline FG (mmol/l)	8.99 (3.89)	7.55 (2.83)	9.27 (3.72)	10.16 (4.44)	< 0.001
Mean 12-month FG	8.10 (2.42)	6.77 (1.67)	7.94 (2.11)	9.55 (2.45)	< 0.001
Mean 24-month FG	8.09 (2.39)	6.83 (1.67)	7.88 (2.11)	9.55 (2.44)	< 0.001
Baseline HbA_1c_ (%)	8.1 (1.9)	7.1 (1.5)	8.1 (1.7)	9.0 (2.1)	< 0.001
(mmol/mol)	65 (20.8)	54 (16.4)	65 (18.6)	75 (23.0)	
Mean 12-month HbA_1c_ (%)	7.8 (1.5)	6.9 (1.0)	7.7 (1.1)	8.9 (1.5)	< 0.001
(mmol/mol)	62 (16.4)	52 (10.9)	61 (12.0)	74 (16.4)	
Mean 24-month HbA_1c_ (%)	7.8 (1.4)	6.9 (0.9)	7.7 (1.1)	8.9 (1.4)	< 0.001
(mmol/mol)	62 (15.3)	52 (9.8)	61 (12.0)	74 (15.3)	
Triacylglycerol (mmol/l)	2.0 (1.5)	1.9 (1.4)	1.9 (1.2)	2.3 (1.8)	0.081
HDL-cholesterol (mmol/l)	1.11 (0.30)	1.11 (0.28)	1.11 (0.28)	1.09 (0.34)	0.47
LDL-cholesterol (mmol/l)	3.03 (1.00)	3.10 (1.01)	2.97 (0.93)	3.05 (1.09)	0.48
Glomerular filtration rate (ml/min/1.73 m^2^)	81 (20)	82 (18)	81 (20)	79 (23)	0.22
Albuminuria (mg/24 h)	13 (7–42)	10 (6–23)	13 (7–28)	20 (9–98)	< 0.001
Macrovascular outcomes^a^					
Total CVEs	128 (2.56)	29 (1.69)	39 (2.38)	60 (3.89)	< 0.001
Major CVEs	96 (1.86)	21 (1.20)	28 (1.67)	47 (2.92)	< 0.001
Cardiovascular mortality	67 (1.26)	14 (0.77)	20 (1.17)	33 (1.94)	0.007
All-cause mortality	158 (2.97)	36 (1.99)	46 (2.69)	76 (4.47)	< 0.001
Microvascular outcomes^b^					
Retinopathy (incident/worsening) (n = 533)	152 (4.88)	32 (2.73)	42 (4.15)	78 (8.84)	< 0.001
Renal composite (n = 615)	183 (4.71)	43 (3.16)	57 (4.64)	83 (6.71)	< 0.001
Microalbuminuria (incident) (n = 436)	89 (3.23)	25 (2.30)	33 (3.61)	31 (4.30)	0.048
Renal failure (n = 615)	91 (2.15)	16 (1.10)	25 (1.85)	50 (3.63)	< 0.001
Peripheral neuropathy (incident/worsening) (n = 471)	96 (20.4%)	20 (12.1%)	34 (22.1%)	42 (28.4%)	< 0.001
Peripheral neuropathy (incident) (n = 338)	42 (12.4%)	13 (9.7%)	13 (12.4%)	16 (16.8%)	0.11

Values are proportions, and means (standard deviations) or medians (interquartile range)

*FG* fasting glycemia, *ACE* angiotensin-converting enzyme, *AR* angiotensin II receptor, *SBP* systolic blood pressure, *DBP* diastolic blood pressure, *HbA*_*1c*_ glycated hemoglobin, *HDL* high-density lipoprotein, *LDL* low-density lipoprotein, CVEs cardiovascular events

^a^Values are absolute numbers (incidence rate per 100 patient-years of follow-up)

^b^Values are absolute numbers (incidence rate per 100 patient-years of follow-up), except for peripheral neuropathy that are absolute numbers (proportions)

### Endpoints occurrence during follow-up

**Fig. 1:**
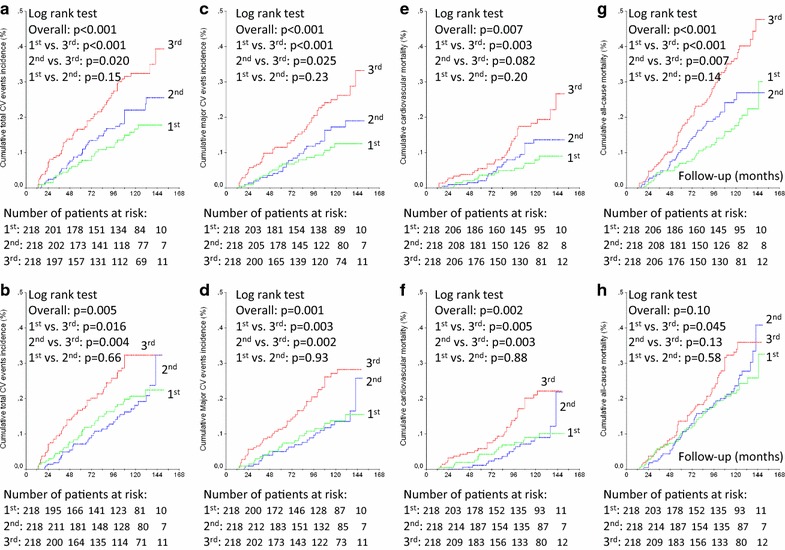
Kaplan-Meier estimates of cumulative incidence of total cardiovascular events (**a**, **b**), major cardiovascular events (MACE, **c**, **d**), cardiovascular deaths (**e**, **f**) and all-cause deaths (**g**, **h**) during follow-up in patients categorized into tertiles (green curve first tertile, blue curve second tertile, and red curve third tertile) of 24-month fasting glycemia standard deviation (upper panels **a**, **c**, **e** and **g**) and HbA_1c_ standard deviation (lower panels **b**, **d**, **f** and **h**)

**Fig. 2:**
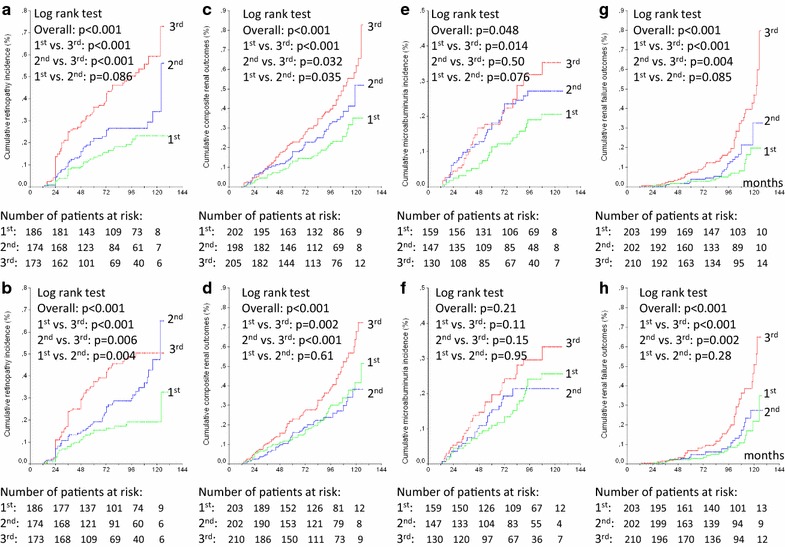
Kaplan–Meier estimates of cumulative diabetic retinopathy incidence or worsening (**a**, **b**), composite renal events (microalbuminuria and renal function deterioration, **c**, **d**), microalbuminuria incidence (**e**, **f**) and renal function deterioration (**g**, **h**) during follow-up in patients categorized into tertiles (green curve first tertile, blue curve second tertile, and red curve third tertile) of 24-month fasting glycemia standard deviation (upper panels **a**, **c**, **e** and **g**) and of 24-month HbA_1c_ standard deviation (lower panels **b**, **d**, **f** and **h**)

Over a median follow-up of 9.3 years (IQR 5.2–10.8 years), 128 patients had a CVE (96 MACE), and 158 patients died (67 from cardiovascular diseases); 152 newly-developed or worsened diabetic retinopathy, 183 achieved the renal composite outcome (89 newly developed microalbuminuria and 91 deteriorated renal function), and 96 newly-developed or worsened peripheral neuropathy. Tables 1 and 2 show that patients with higher long-term glycemic variability had a significantly higher incidence of all endpoints, except of all-cause mortality and new microalbuminuria development for HbA_1c_ variability and of new peripheral neuropathy development for FG variability. Kaplan–Meier curves of cumulative incidence of endpoints (Figs. 1, 2) shows that for most of the endpoints the increased incidence was mainly observed in the highest tertile variability subgroup in relation to the middle and lowest tertile subgroups, except for retinopathy (for HbA_1c_ variability) and renal outcomes (for FG variability), where those patients in the middle tertile subgroup already had an increased incidence of these endpoints.

### Risks associated with increased long-term glycemic variability

Table 3 (for macrovascular and mortality outcomes) and Table 4 (for microvascular outcomes) present the risks associated with a 1-SD increment in each 12- and 24-month glycemic variability parameter after increasing levels of confounding variables adjustments. As a whole, 24-month glycemic variability parameters were better risk predictors than 12-month parameters, and variabilities estimated by SDs and by CVs were roughly equivalent. For cardiovascular endpoints, particularly for MACE, 24-month glycemic variability, either estimated by HbA_1c_ or by FG, were independent predictors of outcomes. However, for all-cause and cardiovascular mortalities, FG variability appeared a stronger risk predictor than HbA_1c_ variability parameters. Regarding microvascular outcomes, no glycemic variability parameter predicted diabetic retinopathy incidence or worsening, which associations were mostly attenuated by mean HbA_1c_ levels. Excluding patients with pre-existent retinopathy (i.e., analyzing separately only new retinopathy development) did not change these results. For renal outcomes, only 24-month FG variability predicted new microalbuminuria development, whereas both HbA_1c_ and FG variabilities independently predicted renal failure outcomes. Excluding patients with nephropathy at baseline (albuminuria ≥ 30 mg/24 h or eGFR < 60 ml/min/1.73 m^2^) also did not change any of the results of renal outcomes. Otherwise, substituting baseline nephropathy by its individual components (albuminuria and eGFR as continuous variables) as adjusting covariates widened the confidence intervals towards non-significant values; although there were only slight reductions in HRs. HbA_1c_ variability predicted new-development of diabetic peripheral neuropathy, but not its worsening. Otherwise, mean HbA_1c_ levels were not predictors of any macrovascular and mortality outcomes, and were only predictors of retinopathy and peripheral neuropathy development or progression, but not of new neuropathy incidence. Tables 5 and 6 show the same analyses with patients categorized into tertiles of glycemic variability parameters, and the results were mainly consistent with the continuous parameters analyses, although some parameters were non-significant because of the wider confidence intervals associated with the lower number of endpoints in each tertile subgroups. There was evidence of interaction (p < 0.10) between mean HbA_1c_ levels and glycemic variability in analyses for diabetic retinopathy. In stratified analyses, 24-month HbA_1c_ variability predicted retinopathy incidence or worsening in individuals with lower HbA_1c_ levels (≤ 7.5%, 58 mmol/mol), with HRs of 1.88 (95% CI 1.03–3.43; p = 0.039) for HbA_1c_-SD and 1.56 (95% CI 1.03–2.36; p = 0.036) for HbA_1c_-VC. Similarly, when divided into tertiles, the upper tertile of HbA_1c_-SD (HR 3.26; 95% CI 1.24–8.52; p = 0.016) and of HbA_1c_-VC (HR 2.70; 95% CI 1.15–6.32; p = 0.023) also predicted retinopathy development or progression. Otherwise, in patients with higher HbA1c levels, no glycemic variability parameter was predictive of retinopathy. There was no evidence of interaction in any of the other outcomes (all p > 0.15 for interaction terms).

**Table 3:** Results of Cox survival analyses for the excess risks associated with 12- and 24-month glycemic variability parameters, analyzed as continuous variables, for the occurrence of future macrovascular complications and mortality

Outcome	Glycemic parameter	12-month glycemic variability			24-month glycemic variability		
		Model 1 HR (95% CI)	Model 2 HR (95% CI)	Model 3 HR (95% CI)	Model 1 HR (95% CI)	Model 2 HR (95% CI)	Model 3 HR (95% CI)
Total CV events n = 128	FG-SD	1.48 (1.28–1.70)*	1.30 (1.10–1.54)^†^	1.23 (1.00–1.51)	1.54 (1.34–1.78)*	1.41 (1.18–1.67)*	1.36 (1.09–1.69)^†^
	FG-VC	1.48 (1.25–1.74)*	1.33 (1.10–1.61)^†^	1.26 (1.04–1.54)^‡^	1.58 (1.33–1.88)*	1.44 (1.18–1.75)*	1.37 (1.12–1.69)^†^
	HbA_1c_-SD	1.33 (1.15–1.53)*	1.22 (1.05–1.42)^†^	1.11 (0.91–1.34)	1.39 (1.21–1.60)*	1.29 (1.11–1.49)^†^	1.20 (1.00–1.44)
	HbA_1c_-VC	1.26 (1.09–1.46)^†^	1.19 (1.03–1.38)^‡^	1.10 (0.93–1.30)	1.32 (1.14–1.52)*	1.25 (1.08–1.44)^†^	1.17 (0.99–1.38)
	HbA_1c_-MEAN^a^	–	–	1.24 (0.96–1.60)	–	–	1.18 (0.93–1.49)
Major CV events n = 96	FG-SD	1.51 (1.28–1.77)*	1.33 (1.09–1.61)^†^	1.19 (0.94–1.51)	1.54 (1.30–1.82)*	1.35 (1.10–1.65)^†^	1.23 (0.96–1.59)
	FG-VC	1.46 (1.20–1.77)*	1.31 (1.05–1.63)^‡^	1.24 (0.99–1.57)	1.53 (1.25–1.87)*	1.34 (1.06–1.69)^‡^	1.29 (1.01–1.64)^‡^
	HbA_1c_-SD	1.40 (1.19–1.63)*	1.28 (1.08–1.51)^†^	1.19 (0.97–1.46)	1.44 (1.23–1.68)*	1.31 (1.11–1.55)^†^	1.23 (1.00–1.51)^‡^
	HbA_1c_-VC	1.34 (1.14–1.57)*	1.25 (1.06–1.47)^†^	1.17 (0.98–1.41)	1.37 (1.17–1.61)*	1.28 (1.08–1.50)^†^	1.21 (1.01–1.44)^‡^
	HbA_1c_-MEAN^a^	–	–	1.14 (0.85–1.53)	–	–	1.16 (0.88–1.52)
CV mortality n = 67	FG-SD	1.51 (1.26–1.82)*	1.47 (1.17–1.86)*	1.29 (0.97–1.73)	1.65 (1.36–2.00)*	1.64 (1.29–2.09)*	1.50 (1.10–2.03)^†^
	FG-VC	1.51 (1.21–1.89)*	1.48 (1.14–1.92)^†^	1.37 (1.04–1.81)^‡^	1.70 (1.34–2.16)*	1.64 (1.25–2.15)*	1.56 (1.17–2.06)^†^
	HbA_1c_-SD	1.51 (1.27–1.80)*	1.38 (1.14–1.67)*	1.24 (0.97–1.58)	1.54 (1.29–1.85)*	1.42 (1.17–1.72)*	1.26 (0.99–1.61)
	HbA_1c_-VC	1.43 (1.20–1.71)*	1.35 (1.12–1.62)^†^	1.23 (1.00–1.52)^‡^	1.44 (1.21–1.73)*	1.37 (1.13–1.65)*	1.24 (1.01–1.54)^‡^
	HbA_1c_-MEAN^a^	–	–	1.28 (0.88–1.84)	–	–	1.27 (0.91–1.79)
All-cause mortality n = 158	FG-SD	1.51 (1.34–1.71)*	1.42 (1.23–1.65)*	1.44 (1.20–1.73)*	1.60 (1.41–1.81)*	1.54 (1.32–1.79)*	1.59 (1.32–1.93)*
	FG-VC	1.53 (1.33–1.77)*	1.45 (1.24–1.71)*	1.43 (1.20–1.69)*	1.66 (1.42–1.93)*	1.55 (1.31–1.85)*	1.53 (1.28–1.82)*
	HbA_1c_-SD	1.41 (1.25–1.60)*	1.29 (1.13–1.48)*	1.25 (1.06–1.47)^†^	1.42 (1.25–1.61)*	1.30 (1.14–1.49)*	1.23 (1.04–1.46)^‡^
	HbA_1c_-VC	1.35 (1.19–1.52)*	1.26 (1.11–1.44)*	1.21 (1.04–1.40)^‡^	1.34 (1.18–1.52)*	1.25 (1.10–1.43)*	1.19 (1.02–1.38)^‡^
	HbA_1c_-MEAN^a^	–	–	1.07 (0.84–1.35)	–	–	1.08 (0.87–1.34)

Values are hazard ratios and 95% confidence intervals, estimated for increases of 1-SD in each glycemic parameter

Model 1 is adjusted for age, sex and number of HbA_1c_ or FG measurements

Model 2 is further adjusted for diabetes duration, BMI, smoking status, physical inactivity, arterial hypertension, number of anti-hypertensive drugs in use, ambulatory 24-h SBP, presence of micro- and macrovascular complications at baseline, serum mean HDL- and LDL-cholesterol, and use of insulin, statins and aspirin

Model 3 is further adjusted for mean fasting glycemia and HbA_1c_

*HR* hazard ratio, *CI* confidence interval, *CV* cardiovascular, *FG-SD* fasting glucose standard deviation, *FG-VC* fasting glucose variation coefficient, *HbA*_*1c*_*-SD* glycated hemoglobin standard deviation, *HbA*_*1c*_*-VC* glycated hemoglobin variation coefficient, *HbA*_*1c*_*-MEAN* mean glycated hemoglobin during the same time interval

* p < 0.001; ^†^ p < 0.01; ^‡^ p < 0.05

^a^The HR of HbA_1c_-MEAN was estimated also for increases of 1-SD in the model with the highest HR of the glycemic variability parameter, whichever it was

**Table 4:** Results of multivariable analyses for the excess risks associated with 12- and 24-month glycemic variability parameters, analyzed as continuous variables, for the occurrence of future diabetic microvascular complications

Outcome	Glycemic parameter	12-month glycemic variability			24-month glycemic variability		
		Model 1 HR (95% CI)	Model 2 HR (95% CI)	Model 3 HR (95% CI)	Model 1 HR (95% CI)	Model 2 HR (95% CI)	Model 3 HR (95% CI)
Retinopathy (incident or worsening) n = 152	FG-SD	1.54 (1.35–1.76)*	1.27 (1.08–1.49)^†^	1.12 (0.93–1.35)	1.49 (1.31–1.69)*	1.28 (1.09–1.51)^†^	1.11 (0.91–1.35)
	FG-VC	1.48 (1.27–1.71)*	1.18 (1.00–1.39)^‡^	1.12 (0.94–1.34)	1.48 (1.27–1.72)*	1.20 (1.01–1.43)^‡^	1.13 (0.93–1.36)
	HbA_1c_-SD	1.27 (1.12–1.43)*	1.16 (1.00–1.33)^‡^	0.99 (0.83–1.18)	1.31 (1.16–1.47)*	1.21 (1.05–1.39)^†^	1.05 (0.89–1.25)
	HbA_1c_-VC	1.21 (1.06–1.38)^†^	1.12 (0.97–1.30)	1.00 (0.85–1.18)	1.27 (1.11–1.44)*	1.17 (1.02–1.34)^‡^	1.06 (0.91–1.24)
	HbA_1c_-MEAN^a^	–	–	1.27 (1.00–1.60)^‡^	–	–	1.27 (1.03–1.58)^‡^
Composite renal outcome n = 183	FG-SD	1.29 (1.14–1.47)*	1.19 (1.03–1.39)^‡^	1.15 (0.97–1.36)	1.37 (1.20–1.55)*	1.30 (1.12–1.51)^†^	1.25 (1.05–1.48)^‡^
	FG-VC	1.24 (1.08–1.42)^†^	1.14 (0.98–1.33)	1.11 (0.94–1.30)	1.30 (1.13–1.49)*	1.21 (1.04–1.41)^‡^	1.19 (1.01–1.40)^‡^
	HbA_1c_-SD	1.24 (1.09–1.40)^†^	1.14 (0.99–1.30)	1.06 (0.90–1.26)	1.29 (1.14–1.46)*	1.19 (1.04–1.37)^‡^	1.12 (0.95–1.32)
	HbA_1c_-VC	1.19 (1.05–1.36)^†^	1.11 (0.97–1.27)	1.05 (0.90–1.22)	1.23 (1.09–1.40)^†^	1.15 (1.01–1.31)^‡^	1.09 (0.94–1.26)
	HbA_1c_-MEAN^a^	–	–	1.15 (0.91–1.46)	–	–	1.12 (0.91–1.39)
Microalbuminuria (incident) n = 89	FG-SD	1.21 (0.98–1.48)	1.24 (0.98–1.56)	1.21 (0.91–1.62)	1.27 (1.04–1.56)^‡^	1.34 (1.06–1.69)^‡^	1.34 (1.00–1.79)^‡^
	FG-VC	1.14 (0.93–1.41)	1.17 (0.93–1.48)	1.15 (0.90–1.48)	1.21 (0.98–1.48)	1.26 (1.00–1.58)^‡^	1.25 (0.98–1.60)
	HbA_1c_-SD	1.13 (0.92–1.40)	1.11 (0.89–1.39)	1.02 (0.76–1.37)	1.20 (0.98–1.47)	1.19 (0.96–1.47)	1.12 (0.84–1.49)
	HbA_1c_-VC	1.11 (0.89–1.37)	1.08 (0.87–1.35)	1.01 (0.78–1.31)	1.17 (0.95–1.43)	1.15 (0.93–1.42)	1.08 (0.85–1.38)
	HbA_1c_-MEAN^a^	–	–	1.03 (0.74–1.45)	–	–	1.00 (0.73–1.37)
Renal failure n = 91	FG-SD	1.54 (1.31–1.83)*	1.27 (1.05–1.55)^‡^	1.22 (0.98–1.53)	1.70 (1.44–2.01)*	1.43 (1.16–1.77)^†^	1.37 (1.08–1.74)^†^
	FG-VC	1.50 (1.24–1.82)*	1.25 (1.01–1.53)^‡^	1.18 (0.95–1.46)	1.64 (1.34–2.00)*	1.32 (1.06–1.64)^‡^	1.29 (1.02–1.61)^‡^
	HbA_1c_-SD	1.51 (1.29–1.76)*	1.34 (1.12–1.60)^†^	1.27 (1.02–1.58)^‡^	1.53 (1.30–1.79)*	1.35 (1.12–1.63)^†^	1.25 (1.00–1.60)^‡^
	HbA_1c_-VC	1.43 (1.22–1.68)*	1.33 (1.11–1.59)^†^	1.26 (1.03–1.53)^‡^	1.43 (1.22–1.68)*	1.31 (1.09–1.57)^†^	1.23 (1.01–1.50)^‡^
	HbA_1c_-MEAN^a^	–	–	1.20 (0.85–1.70)	–	–	1.28 (0.96–1.70)
Peripheral neuropathy (incident or worsening) n = 96	FG-SD	1.37 (1.10–1.71)^†^	1.09 (0.82–1.44)	0.96 (0.69–1.34)	1.58 (1.25–2.00)*	1.31 (0.98–1.76)	1.21 (0.86–1.70)
	FG-VC	1.38 (1.10–1.73)^†^	1.16 (0.87–1.54)	1.04 (0.77–1.40)	1.56 (1.24–1.96)*	1.38 (1.04–1.83)^‡^	1.25 (0.93–1.68)
	HbA_1c_-SD	1.42 (1.15–1.76)^†^	1.30 (1.01–1.66)^‡^	1.11 (0.82–1.50)	1.51 (1.21–1.87)*	1.37 (1.07–1.75)^‡^	1.17 (0.86–1.60)
	HbA_1c_-VC	1.33 (1.07–1.65)^‡^	1.22 (0.95–1.55)	1.08 (0.83–1.42)	1.41 (1.14–1.74)^†^	1.26 (0.99–1.61)	1.12 (0.86–1.47)
	HbA_1c_-MEAN^a^	–	–	1.55 (1.03–2.32)^‡^	–	–	1.53 (1.09–2.15)^‡^
Peripheral neuropathy (incident) n = 42	FG-SD	1.33 (0.96–1.84)	1.10 (0.75–1.63)	0.95 (0.60–1.50)	1.44 (1.03–2.01)^‡^	1.23 (0.83–1.84)	1.09 (0.68–1.74)
	FG-VC	1.26 (0.91–1.75)	1.12 (0.76–1.65)	1.03 (0.68–1.55)	1.31 (0.95–1.82)	1.20 (0.81–1.77)	1.12 (0.74–1.69)
	HbA_1c_-SD	1.66 (1.27–2.17)*	1.63 (1.19–2.22)^†^	1.73 (1.15–2.60)^†^	1.76 (1.33–2.32)*	1.67 (1.22–2.27)*	1.82 (1.20–2.75)^†^
	HbA_1c_-VC	1.63 (1.25–2.14)*	1.58 (1.16–2.16)^†^	1.55 (1.09–2.20)^‡^	1.72 (1.30–2.26)*	1.62 (1.18–2.21)^†^	1.60 (1.12–2.28)^†^
	HbA_1c_-MEAN^a^	–	–	0.91 (0.47–1.74)	–	–	0.85 (0.47–1.57)

Values are hazard ratios and 95% confidence intervals, estimated by Cox analyses for increases of 1-SD in each glycemic parameter; except for peripheral neuropathy outcomes that are odds ratios and 95% confidence intervals, estimated by logistic regressions

Models 1, 2 and 3 were adjusted for the same covariates as in Table 3, except for peripheral neuropathy that was adjusted for height instead of BMI and further for the time interval between baseline and second neuropathy examination

*HR* hazard ratio, *CI* confidence interval, *FG-SD* fasting glucose standard deviation, *FG-VC* fasting glucose variation coefficient, *HbA*_*1c*_*-SD* glycated hemoglobin standard deviation, *HbA*_*1c*_*-VC* glycated hemoglobin variation coefficient, *HbA*_*1c*_*-MEAN* mean glycated hemoglobin during the same time interval

* p < 0.001; ^†^ p < 0.01; ^‡^ p < 0.05

^a^The HR of HbA_1c_-MEAN was estimated also for increases of 1-SD in the model with the highest HR of the glycemic variability parameter, whichever it was

**Table 5:** Results of Cox survival analyses for the excess risks associated with 12- and 24-month glycemic variability parameters, divided into tertiles, for the occurrence of future macrovascular complications and mortality

Outcome	Glycemic parameter	12-month glycemic variability			24-month glycemic variability		
		Model 1 HR (95% CI)	Model 2 HR (95% CI)	Model 3 HR (95% CI)	Model 1 HR (95% CI)	Model 2 HR (95% CI)	Model 3 HR (95% CI)
Total CV events n = 128	FG-SD	2.85 (1.79–4.54)*	2.08 (1.22–3.54)^†^	1.71 (0.94–3.10)	2.39 (1.52–3.77)*	1.60 (0.95–2.72)	1.22 (0.68–2.21)
	FG-VC	2.35 (1.49–3.68)*	1.82 (1.10–3.01)^‡^	1.62 (0.97–2.71)	2.54 (1.62–3.99)*	1.97 (1.18–3.31)^‡^	1.76 (1.04–3.00)^‡^
	HbA_1c_-SD	1.93 (1.26–2.95)^†^	1.44 (0.91–2.28)	1.04 (0.59–1.82)	1.87 (1.22–2.87)^†^	1.43 (0.90–2.25)	1.03 (0.59–1.79)
	HbA_1c_-VC	1.76 (1.15–2.71)^‡^	1.47 (0.94–2.29)	1.18 (0.72–1.92)	2.01 (1.31–3.11)^†^	1.69 (1.07–2.66)^‡^	1.38 (0.84–2.26)
Major CV events n = 96	FG-SD	2.74 (1.63–4.61)*	1.83 (1.01–3.32)^‡^	1.37 (0.70–2.68)	2.64 (1.56–4.48)*	1.64 (0.89–3.03)	1.24 (0.63–2.44)
	FG-VC	1.92 (1.16–3.19)^‡^	1.42 (0.81–2.49)	1.31 (0.73–2.33)	2.37 (1.42–3.97)^†^	1.69 (0.94–3.05)	1.54 (0.85–2.80)
	HbA_1c_-SD	2.47 (1.51–4.05)*	1.73 (1.03–2.93)^‡^	1.39 (0.75–2.57)	2.42 (1.47–3.99)^†^	1.67 (0.98–2.86)	1.30 (0.70–2.43)
	HbA_1c_-VC	2.17 (1.34–3.51)^†^	1.71 (1.04–2.82)^‡^	1.43 (0.83–2.44)	2.33 (1.43–3.79)^†^	1.78 (1.07–2.97)^‡^	1.49 (0.86–2.57)
CV mortality n = 67	FG-SD	3.26 (1.73–6.13)*	2.67 (1.28–5.59)^†^	1.85 (0.81–4.24)	3.27 (1.72–6.22)*	2.50 (1.17–5.33)^‡^	1.72 (0.75–3.96)
	FG-VC	2.26 (1.20–4.24)^‡^	1.92 (0.94–3.94)	1.60 (0.76–3.37)	3.23 (1.68–6.21)*	2.82 (1.34–5.95)^†^	2.41 (1.13–5.17)^‡^
	HbA_1c_-SD	3.18 (1.77–5.71)*	2.60 (1.38–4.91)^†^	2.07 (0.97–4.40)	3.02 (1.65–5.51)*	2.40 (1.25–4.62)^†^	1.72 (0.80–3.71)
	HbA_1c_-VC	2.74 (1.53–4.89)^†^	2.22 (1.22–4.06)^†^	1.78 (0.92–3.44)	2.81 (1.57–5.02)*	2.33 (1.26–4.32)^†^	1.81 (0.93–3.54)
All-cause mortality n = 158	FG-SD	3.20 (2.14–4.79)*	2.62 (1.64–4.17)*	2.60 (1.55–4.37)*	2.80 (1.86–4.21)*	2.05 (1.28–3.27)^†^	1.82 (1.09–3.06)^‡^
	FG-VC	2.45 (1.63–3.69)*	2.07 (1.31–3.28)^†^	1.96 (1.22–3.14)^†^	2.71 (1.79–4.08)*	2.21 (1.39–3.51)*	2.06 (1.28–3.31)^†^
	HbA_1c_-SD	2.15 (1.45–3.20)*	1.64 (1.07–2.51)^‡^	1.35 (0.80–2.25)	1.95 (1.31–2.91)*	1.48 (0.96–2.28)	1.10 (0.66–1.83)
	HbA_1c_-VC	2.12 (1.43–3.15)*	1.72 (1.15–2.59)^†^	1.51 (0.97–2.35)	2.19 (1.46–3.27)*	1.76 (1.16–2.69)^†^	1.49 (0.95–2.34)

Values are hazard ratios for the highest tertile subgroup in relation to the lowest one and 95% confidence intervals

Model 1 is adjusted for age, sex and number of HbA_1c_ or FG measurements

Model 2 is further adjusted for diabetes duration, BMI, smoking status, physical inactivity, arterial hypertension, number of anti-hypertensive drugs in use, ambulatory 24-h SBP, presence of micro- and macrovascular complications at baseline, serum mean HDL- and LDL-cholesterol, and use of insulin, statins and aspirin

Model 3 is further adjusted for mean fasting glycemia and HbA_1c_

*HR* hazard ratio, *CI* confidence interval, *CV* cardiovascular, *FG-SD* fasting glucose standard deviation, *FG-VC* fasting glucose variation coefficient, *HbA*_*1c*_*-SD* glycated hemoglobin standard deviation, *HbA*_*1c*_*-VC* glycated hemoglobin variation coefficient

* p < 0.001; ^†^ p < 0.01; ^‡^ p < 0.05

**Table 6:** Results of multivariable analyses for the excess risks associated with 12- and 24-month glycemic variability parameters, divided into tertiles, for the occurrence of future diabetic microvascular complications

Outcome	Glycemic parameter	12-month glycemic variability			24-month glycemic variability		
		Model 1 HR (95% CI)	Model 2 HR (95% CI)	Model 3 HR (95% CI)	Model 1 HR (95% CI)	Model 2 HR (95% CI)	Model 3 HR (95% CI)
Retinopathy (incident or worsening) n = 152	FG-SD	2.78 (1.84–4.21)*	1.66 (1.04–2.65)^‡^	1.17 (0.69–1.98)	3.24 (2.14–4.92)*	1.89 (1.18–3.02)^†^	1.36 (0.79–2.33)
	FG-VC	2.47 (1.64–3.71)*	1.50 (0.96–2.33)	1.31 (0.82–2.09)	2.47 (1.64–3.70)*	1.45 (0.93–2.25)	1.23 (0.77–1.96)
	HbA_1c_-SD	2.59 (1.70–3.94)*	1.68 (1.07–2.64)^‡^	1.13 (0.66–1.95)	3.02 (1.96–4.65)*	2.01 (1.26–3.19)^†^	1.44 (0.84–2.46)
	HbA_1c_-VC	2.03 (1.34–3.06)^†^	1.50 (0.98–2.31)	1.16 (0.74–1.84)	2.47 (1.62–3.76)*	1.82 (1.17–2.84)^†^	1.41 (0.88–2.26)
Composite renal outcome n = 183	FG-SD	1.87 (1.29–2.71)^†^	1.52 (1.00–2.31)	1.32 (0.82–2.13)	2.18 (1.50–3.17)*	1.88 (1.23–2.87)^†^	1.66 (1.03–2.67)^‡^
	FG-VC	1.69 (1.17–2.45)^†^	1.49 (0.99–2.22)	1.39 (0.91–2.12)	1.84 (1.28–2.66)^†^	1.66 (1.12–2.51)^‡^	1.60 (1.05–1.43)^‡^
	HbA_1c_-SD	1.52 (1.06–2.17)^‡^	1.23 (0.84–1.81)	1.00 (0.62–1.59)	1.68 (1.18–2.39)^†^	1.38 (0.94–2.02)	1.14 (0.72–1.81)
	HbA_1c_-VC	1.42 (1.00–2.02)^‡^	1.22 (0.85–1.76)	1.06 (0.72–1.58)	1.56 (1.10–2.21)^‡^	1.35 (0.93–1.94)	1.16 (0.79–1.72)
Microalbuminuria (incident) n = 89	FG-SD	1.66 (0.98–2.81)	1.67 (0.93–2.98)	1.60 (0.81–3.13)	1.70 (1.00–2.90)^‡^	1.78 (0.98–3.22)	1.64 (0.81–3.33)
	FG-VC	1.32 (0.78–2.25)	1.37 (0.77–2.42)	1.31 (0.73–2.37)	1.63 (0.96–2.78)	1.85 (1.03–3.30)^‡^	1.80 (0.98–3.30)
	HbA_1c_-SD	1.10 (0.66–1.83)	1.04 (0.60–1.81)	0.80 (0.40–1.60)	1.31 (0.79–2.18)	1.28 (0.74–2.22)	1.06 (0.54–2.11)
	HbA_1c_-VC	1.09 (0.65–1.81)	1.05 (0.62–1.79)	0.90 (0.50–1.63)	1.32 (0.79–2.19)	1.27 (0.74–2.19)	1.09 (0.59–2.01)
Renal failure n = 91	FG-SD	2.70 (1.54–4.72)*	1.70 (0.90–3.20)	1.36 (0.67–2.77)	3.63 (2.05–6.45)*	2.14 (1.13–4.07)^‡^	1.75 (0.86–3.54)
	FG-VC	2.56 (1.46–4.50)*	1.86 (1.01–3.44)^‡^	1.63 (0.86–3.08)	2.67 (1.56–4.59)*	1.71 (0.95–3.09)	1.57 (0.85–2.88)
	HbA_1c_-SD	3.40 (1.95–5.92)*	2.34 (1.29–4.24)^†^	2.12 (1.05–4.27)^‡^	3.12 (1.82–5.37)*	2.13 (1.19–3.81)^‡^	1.78 (0.91–3.49)
	HbA_1c_-VC	2.43 (1.43–4.11)*	1.91 (1.10–3.30)^‡^	1.66 (0.92–2.98)	2.38 (1.42–4.01)*	1.78 (1.03–3.08)^‡^	1.50 (0.84–2.67)
Peripheral neuropathy (incident or worsening) n = 96	FG-SD	2.50 (1.35–4.62)^†^	1.45 (0.70–3.00)	1.05 (0.46–2.41)	2.82 (1.54–5.13)*	1.73 (0.85–3.50)	1.35 (0.60–3.03)
	FG-VC	3.45 (1.77–6.72)*	2.52 (1.17–5.39)^‡^	2.02 (0.91–4.45)	3.60 (1.91–6.79)*	2.82 (1.36–5.87)^†^	2.33 (1.09–4.97)^‡^
	HbA_1c_-SD	2.07 (1.16–3.77)^‡^	1.35 (0.69–2.68)	0.76 (0.33–1.75)	2.14 (1.18–3.90)^‡^	1.39 (0.70–2.77)	0.79 (0.34–1.79)
	HbA_1c_-VC	1.82 (1.00–3.34)^‡^	1.34 (0.69–2.62)	0.97 (0.47–2.01)	2.38 (1.29–4.39)^†^	1.63 (0.83–3.22)	1.20 (0.57–2.50)
Peripheral neuropathy (incident) n = 42	FG-SD	2.04 (0.90–4.62)	1.46 (0.54–3.95)	0.99 (0.31–3.16)	1.78 (0.80–3.97)	1.15 (0.44–2.99)	0.73 (0.24–2.25)
	FG-VC	2.56 (1.07–6.13)^‡^	2.13 (0.78–5.81)	1.80 (0.64–5.07)	2.46 (1.05–5.78)^‡^	2.18 (0.83–5.72)	1.88 (0.69–5.13)
	HbA_1c_-SD	2.17 (0.98–4.79)	1.55 (0.62–3.85)	1.07 (0.35–3.33)	2.21 (1.00–4.89)^‡^	1.59 (0.64–3.94)	1.15 (0.37–3.52)
	HbA_1c_-VC	2.78 (1.16–6.67)^‡^	2.14 (0.82–5.58)	1.73 (0.61–4.94)	3.06 (1.28–7.23)^‡^	2.22 (0.86–5.75)	1.85 (0.66–5.18)

Values are hazard ratios and 95% confidence intervals, estimated by Cox analyses, for the highest tertile subgroup in relation to the lowest one; except for peripheral neuropathy outcomes that are odds ratios and 95% confidence intervals, estimated by logistic regressions

Models 1, 2 and 3 were adjusted for the same covariates as in Table 3, except for peripheral neuropathy that was adjusted for height instead of BMI and further for the time interval between baseline and second neuropathy examination

*HR* hazard ratio, *CI* confidence interval, *CV* cardiovascular, *FG-SD* fasting glucose standard deviation, *FG-VC* fasting glucose variation coefficient, *HbA*_*1c*_*-SD* glycated hemoglobin standard deviation, *HbA*_*1c*_*-VC* glycated hemoglobin variation coefficient

* p < 0.001; ^†^ p < 0.01; ^‡^ p < 0.05

## Discussion

This prospective cohort study with a median follow-up of nearly 10 years has some important new findings. First, it demonstrated that for all micro- and macrovascular outcomes, except for retinopathy and peripheral neuropathy development or progression, 24-month visit-to-visit glycemic variability parameters, either estimated for HbA_1c_ or for FG, were better risk predictors than mean HbA_1c_ levels during this same time interval. Second, specifically for diabetic retinopathy development or progression, 24-month HbA_1c_ variability was a significant risk predictor in patients with good glycemic control (with mean HbA1c ≤ 7.5%, 58 mmol/mol); but not in those with poorer controlled diabetes (HbA1c > 7.5%), where mean HbA_1c_ levels were the main risk predictor. Third, specifically for peripheral neuropathy, mean HbA_1c_ was the main risk predictor for the composite outcome of developing or worsening neuropathy, whereas HbA_1c_ variability was a better risk predictor for new-incident peripheral neuropathy. Overall, our findings support the concept that long-term visit-to-visit glycemic variability is an additional and frequently a better glycemic parameter than mean HbA_1c_ levels for assessing the risk of future development of micro- and macrovascular diabetic complications.

Several previous studies evaluated long-term glycemic variability parameters in patients with type 2 diabetes [13–41]. As a whole, most agree that glycemic variability predicts all-cause mortality [29, 32, 33, 36–40], fatal or non-fatal cardiovascular diseases [23, 28–32], new microalbuminuria development [16, 18–22, 25] and renal function deterioration [16, 23, 24, 26], although there were opposing reports for these outcomes [19, 29, 33–36, 41]. A recent meta-analysis including studies published until 2014 confirmed these findings [12]. Our results support these previous investigations. An intriguing finding of our study was that, mainly for cardiovascular and all-cause mortalities, FG variability seemed a stronger risk predictor than HbA_1c_ variability. The reason can be simple statistical adjustments because mean HbA_1c_ is expected to attenuate more HbA_1c_ variability than FG variability. However, models were also adjusted for mean FG levels that would attenuate FG variability, although not at the same extent given that mean HbA_1c_ was more strongly associated with the outcomes than mean FG. Alternatively, FG variability may have captured more accurately hypoglycemic episodes than HbA_1c_ variability [36, 48]. Severe hypoglycemia is well-known associated with adverse prognosis in type 2 diabetes, particularly with increased mortality [49, 50]. Postprandial hyperglycemia is another issue of concern that might not have been adequately captured by either FG or HbA_1c_ variability parameters. In this regard, low 1,5-anhydroglucitol levels, a potential marker of postprandial hyperglycemia, have been reported to predict worse cardiovascular outcomes in patients with acute coronary syndrome [51] and in patients with stable coronary heart disease submitted to elective angiography [52], both groups with low HbA_1c_ levels (< 7.0%).

Regarding the value of glycemic variability as risk predictor for future diabetic retinopathy development or progression, previous reports were controversial, with some showing the predictive capacity of FG variability [13, 15], whereas others negated the importance of glycemic variability parameters [14, 16–18]. The recent meta-analysis also did not demonstrate any prognostic value for retinopathy development [12], but included only two studies. Our study provided new findings, by showing that the predictive power of HbA_1c_ variability for retinopathy development or progression depends on mean HbA_1c_ levels, being positive in patients with better-controlled diabetes, but absent in those poorly-controlled.

As far as we know, this is the first prospective study to assess the importance of glycemic variability for predicting diabetic peripheral neuropathy development or progression. Only a previous study [27] had evaluated its value for cardiovascular autonomic neuropathy development, with positive findings. We showed that HbA_1c_ variability was a predictor mainly of new-incident peripheral neuropathy, whereas mean HbA_1c_ levels mainly predicted its worsening.

From a physiopathological standpoint, there were several potential mechanisms that may link increased glycemic variability to the future occurrence of diabetic micro- and macrovascular complications and to mortality. Acute, short-term glycemia fluctuations induce superoxide overproduction, increased oxidative stress, inflammatory cytokines generation and endothelial dysfunction and damage [11, 53, 54], all linked to chronic diabetic complications. Moreover, exaggerated glycemic fluctuations were demonstrated to adversely affect endothelial vessel healing, increasing neointimal thickness following percutaneous stent implantation [55], and augmenting the risk of periprocedural and short-term cardiovascular complications [56, 57], which may be particularly important in such a high cardiovascular risk population as ours. Further, transient hyperglycemia might cause epigenetic changes, inducing cellular metabolic memory [58, 59], increasing insulin resistance [60] and pancreatic β-cell dysfunction and apoptosis [61]. Alternatively, but not excluding, increased glycemic variability might simply be a marker of unstable glycemic control due to poor treatment adherence and self-management patient compliance [12, 31], multimorbidity, poor quality of life and lack of social support, and frequent infections complications [12]. In this regard, it should be noted that patients with higher glycemic variability at baseline were less physically active, more frequently current or past smokers, and had a greater prevalence of diabetic complications than those with lower variability, particularly evident for FG variability.

The study has some limitations that shall be noted. First, it is a prospective observational cohort; hence no cause-and-effect relations, nor physiopathological inferences, can be made, but only speculated. Moreover, as with any cohort study, residual confounding due to unmeasured or unknown factors can not be ruled out. Second, it enrolled mainly middle-aged to elderly individuals with long-standing type 2 diabetes and with a high prevalence of chronic complications followed-up in a tertiary-care university hospital. Hence, our results might not be generalized to younger individuals with recent onset type 2 diabetes or at primary care management. Third, changes in anti-diabetic medications during follow-up, particularly initiating or increasing insulin dosages, which probably affected glycemic variability during the first 2 years of follow-up, were not taken into account. Forth, peripheral neuropathy assessment was not performed annually during follow-up, as the other outcomes, but on two specific time points (at baseline and after a median of 6 years), which might have affected this endpoint evaluation, although this specific analysis took into account the differential time interval between neuropathy assessments. Finally, we did not adjust for multiple comparisons within each outcome. However, as we have evaluated 4 glycemic variability parameters obtained during 2 time intervals, that is 8 measures for each outcome; if we applied Bonferroni’s correction, we would have considered a p value < 0.006 as significant. With this more conservative approach, only the predictive capacity of glycemic variability for MACE and microalbuminuria incidence would be lost. On the other hand, this study main strength is its well-documented cohort with standardized care and annual outcomes evaluation over a long follow-up, which permitted the most comprehensive analysis of the associations between long-term glycemic variability parameters and risks of separate micro- and macrovascular complications and of mortality in patients with type 2 diabetes.

## Conclusions

This prospective cohort study with a long follow-up of high cardiovascular risk individuals with type 2 diabetes provides evidence that 24-month visit-to-visit HbA_1c_ and FG variabilities are better risk predictors than mean HbA_1c_ levels for all micro- and macrovascular complications and all-cause mortality outcomes, except for retinopathy development/progression in patients with poorly-controlled diabetes and for peripheral neuropathy progression. Reducing glycemic variability can be achieved [62]; however, whether this reduction would translate into better prognosis in patients with diabetes, it still remains to be demonstrated. A single randomized trial [63] in post-myocardial infarction patients with type 2 diabetes did not demonstrated any benefit for cardiovascular outcomes, although the differences in glycemic variability (mainly postprandial glycemia) between the two groups were small, which precluded the demonstration of any benefit [42]. More randomized trials on this issue are needed before we may recommend moving from mean HbA_1c_ levels to glycemic variability as the main parameter of glycemic control monitoring. However, until then, glycemic variability parameter shall at least be measured as an additional parameter to improve risk stratification in patients with type 2 diabetes.

## Abbreviations

ABPM: ambulatory blood pressure monitoring
BMI: body mass index
BP: blood pressure
CI: confidence interval
CVE: cardiovascular events
DBP: diastolic blood pressure
eGFR: estimated glomerular filtration rate
FG: fasting glycaemia
HbA_1c_: glycated hemoglobin
HR: hazard ratio
MACE: major adverse cardiovascular events
OR: odds ratio
SBP: systolic blood pressure
SD: standard deviation
VC: variation coefficient
